# An Integrated Multi-Sensor Information System for Real-Time Reservoir Monitoring and Management

**DOI:** 10.3390/s25185730

**Published:** 2025-09-14

**Authors:** Shiwei Shao, Fan Zhou, Yuxuan Wang, Jiawei Wu

**Affiliations:** 1 Wuhan Vocational College of Software and Engineering, Wuhan Open University, Wuhan 430074, China; 2 Hubei Engineering Research Center for Intelligent Detection and Identification of Complex Parts, Wuhan 430074, China; 3 College of Information Engineering, Shanghai Maritime University, Shanghai 201306, China; fanzhou_cv@163.com (F.Z.); 202430310221@stu.shmtu.edu.cn (Y.W.); 202430310108@stu.shmtu.edu.cn (J.W.); 4 Shanghai Engineering Research Center of Ship Exhaust Intelligent Monitoring, Shanghai 201306, China

**Keywords:** reservoir management, multi-sensor data fusion, heterogeneous data integration, closed-loop control system, decision support system

## Abstract

Reservoirs face growing challenges in safety and sustainable management, requiring systematic approaches that integrate monitoring, analysis, and decision support. To address this need, this study develops an integrated information system framework with a four-layer architecture, encompassing “perception,” “data,” “model,” and “application.” The perception layer establishes a multi-platform monitoring network based on fused multi-sensor data. The data layer manages heterogeneous information through correlation mechanisms at the physics, semantics, and application levels. The model layer supports decision-making through a cross-coupled analytical framework for the coordinated management of water safety, resources, environment, and ecology. Finally, the application layer utilizes virtual-physical mapping and dynamic reasoning to implement a closed-loop management system encompassing forecasting, warning, simulation, and planning. This framework was implemented and validated at the Ye Fan Reservoir in Hubei Province, China. By integrating components like “One Map,” flood dispatching, safety monitoring, early warning, video surveillance, and operational supervision, a three-dimensional perception network was constructed. This deployment significantly improved the precision, reliability, and scientific basis of reservoir operation. The integrated monitoring and management system presented in this paper, driven by heterogeneous sensor networks, provides an effective and generalizable solution for modern reservoir management, with the potential for extension to broader water resource and infrastructure systems.

## 1. Introduction

Global climate change has increased the frequency and intensity of extreme hydrological events [1]. The 158th bulletin of the International Commission on Large Dams states that seepage, displacement, cracks, and erosion are key indicators for monitoring tailings dams and earth-rock dams. The bulletin emphasizes that traditional visual inspections often fail to detect these issues in a timely manner, thus recommending the use of advanced monitoring technologies [2]. This exposes fundamental flaws in the traditional water engineering management paradigm, which relies on static models based on two-dimensional drawings and regular manual inspections. Such methods cannot capture the dynamic interactions between the dam, reservoir, geology, and environment, nor can they support real-time decision-making in complex scenarios like flood regulation [3,4]. The partial breach of the Annamaya reservoir in Andhra Pradesh, India, in 2021 indicates that fragmented data from multiple sources, such as remote sensing images, seepage monitoring, and geological exploration, are the core bottlenecks hindering risk prediction [5].

Digital twin technology offers new opportunities to solve the aforementioned problems [6]. This technology works by establishing a two-way data mapping between physical entities and virtual models. It has been widely applied in numerous fields ranging from industrial manufacturing to transportation [7,8]. However, the application of digital twins in intelligent water management is still relatively rare. In terms of spatial scale, the geometric size and geographical complexity of reservoir dams far exceed those of industrial equipment. High-precision three-dimensional reconstruction requires coordinated cross-scale modeling of terrain and landform at the meter level and reservoir structure details at the millimeter level [9]. In terms of time scale, the lack of standardized protocols for integrating real-time monitoring data, such as seepage and displacement, with static Building Information Modeling (BIM)/Geographic Information System (GIS) models can lead to the “data island” effect [10]. Although there have been studies proposing a general theoretical framework for digital twins [7,11], a systematic methodology for engineering implementation in reservoir scenarios is still lacking.

The actual deployment of digital twin technology is highly dependent on the dynamic integration of multi-dimensional and multi-modal data [12,13]. However, this process faces multiple technical bottlenecks in the application of water conservancy. First, the spatio-temporal heterogeneity of multi-source heterogeneous data significantly increases the complexity of data fusion. In the water conservancy engineering scenario, the collection frequency and format of monitoring data are highly differentiated [10]. For example, Global Navigation Satellite System (GNSS) displacement sensors generate structured time-series data at a second-level frequency, while the TB-level point cloud data generated by unmanned aerial vehicle oblique photography needs to be updated on a weekly or monthly basis. Additionally, the streaming data output by sensors such as seepage pressure sensors and radar water level meters coexist with unstructured text reports from manual inspections. This spatio-temporal fragmentation leads to the need for a large amount of computing power for data alignment. Second, the fragmentation of cross-domain data semantics further exacerbates the difficulty of integration [14]. Water conservancy, meteorological, and geological departments adopt differentiated data encoding systems. For example, a single water level parameter might be labeled as “WaterLevel” (international standard), “WL” (industry abbreviation), or even use a non-English encoding. Furthermore, the random switching of unit dimensions between meters, centimeters, and feet adds another layer of complexity. These inconsistencies cause significant confusion during model input.

Moreover, the development of sophisticated management systems for reservoirs, often envisioned as digital twins, faces a significant implementation gap. While the concept promises a unified view of operations, current initiatives mostly prioritize high-fidelity modeling and visualization over solving core business challenges such as integrated flood control, operational safety, and ecological management [15,16,17]. This “technology–business” gap often stems from a lack of a robust, integrated foundation for data acquisition and processing.

To bridge this gap and lay the groundwork for future advanced applications, this study develops an integrated monitoring and information system framework structured into four layers. The perception layer establishes a three-dimensional, multi-platform collaborative monitoring network that integrates satellite, unmanned aerial vehicle (UAV), in situ, and underwater sensors, enabling comprehensive data acquisition. The data layer employs a multi-level correlation mechanism to fuse this diverse, heterogeneous sensor data into a coherent information model. The model layer then leverages this integrated data to support a cross-coupled analytical framework, incorporating hydrological, structural, and environmental models for multi-objective decision support. Finally, the application layer closes the loop by linking data-driven forecasting, early warning, simulation, and planning directly to operational workflows. This architecture is not a full digital twin, but rather the critical data and system foundation upon which one can be built. Its successful implementation at the Ye Fan Reservoir demonstrates the practicality of this approach and its potential as a scalable precursor to more complex, model-driven systems.

## 2. Materials and Methods

**Figure 1:**
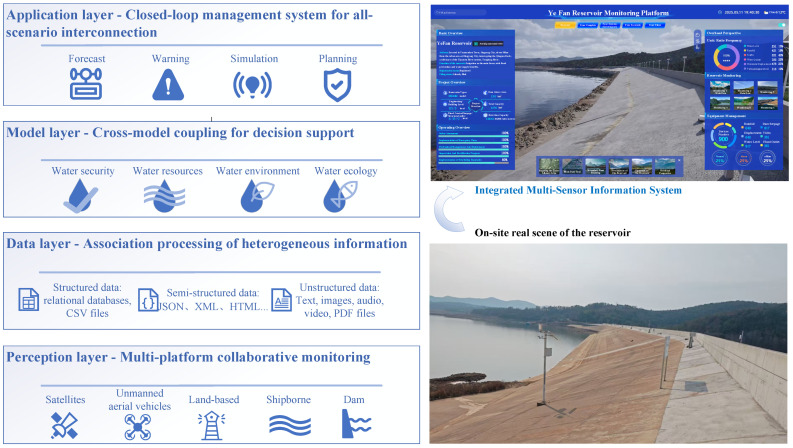
Architectural diagram of the integrated reservoir monitoring and information system framework.

Reservoir management is confronted with several core issues, such as fragmented monitoring systems (relying on a single platform leads to data islands), difficulty in integrating multi-source heterogeneous data (disconnection between physical attributes and business semantics), isolation of decision-making models (separation of water safety, resources, environment and other goals), and weak dynamic simulation capabilities (relying on static plans). These issues result in frequent monitoring blind spots, inefficient cross-departmental collaboration, insufficient global optimization, and delayed disaster response, leading to systematic shortcomings. Therefore, this study proposes an integrated reservoir monitoring and information system framework, structured around a four-layer “perception-data-model-application” architecture, as shown in Figure 1.

The integrated reservoir monitoring and management framework employs a hierarchical architecture, structured around four core components: perception, data, model, and application layers. The system establishes a seamless workflow from data acquisition to operational execution, creating an integrated data-to-decision pipeline. The perception layer serves as the basic support, achieving a comprehensive and dynamic perception of the physical space of the reservoir through multi-platform collaborative observation, including satellites, unmanned aircraft, shore-based, underwater, and the dam body. The data layer processes the heterogeneous information obtained by the perception layer. It covers structured data, semi-structured data, and unstructured data, providing multi-dimensional data fusion support for the upper-layer models. The model layer utilizes cross-model coupling technology to develop four intelligent decision-making sub-models. Through multi-objective collaborative optimization and dynamic simulation, it realizes comprehensive analysis and risk assessment in complex scenarios. The application layer employs a progressive closed-loop management mechanism, “forecasting-warning-simulation-planning.” It converts model outputs into actual business applications. This supports the observation and prediction of reservoir operation status, accident warning, scenario rehearsal, and plan generation, ultimately forming a comprehensive management closed-loop system with full-scenario interconnection.

### 2.1. Multi-Platform Collaborative Monitoring

**Figure 2:**
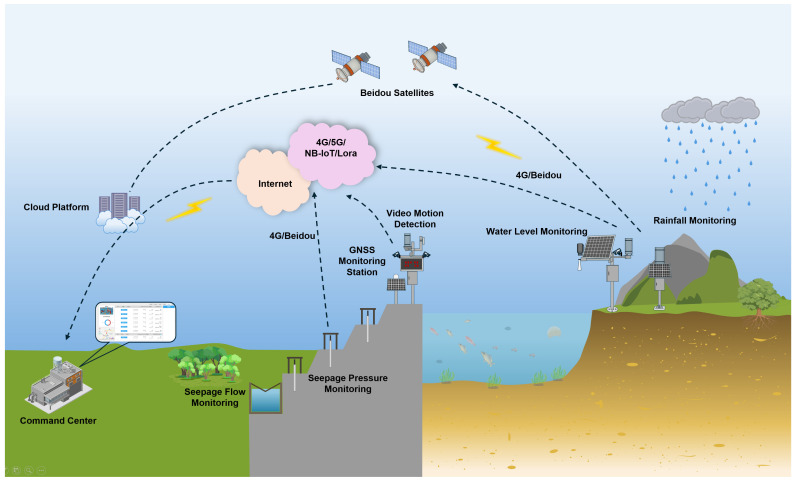
Multi-platform collaborative observation of the reservoir.

The safe operation and intelligent management of reservoirs require multi-platform collaborative observation. To meet this need, this study constructed a three-dimensional monitoring network. This integrates satellites, unmanned aerial vehicles, land-based systems, shipborne equipment, and sensors on the dam body. Each platform offers unique technical advantages and spatial coverage. Together, they form a comprehensive observation system (Figure 2). This system features complementary functions, progressive scales, and integrated data.

#### 2.1.1. Satellite Platform

The satellite platform provides wide-area and periodic coverage [18]. It utilizes several key technologies to achieve this. These include high/multi-spectral optical remote sensing, synthetic aperture radar (SAR), meteorological satellites, and the Beidou Satellite Navigation System (BDS). This platform enables large-scale and macroscopic situational awareness. It monitors the surface characteristics of the entire river basin (such as land use/cover). It tracks the dynamics of rivers and lakes, including changes in area and turbidity. The system also assesses flood disasters by mapping their inundation range. Finally, it observes large-scale water projects, like the overall shape of a reservoir area.

#### 2.1.2. UAV Platform

The UAV platform provides flexible and mobile monitoring with a quick response time. UAVs can be equipped with various payloads. These include LiDAR, small weather radars, high-resolution optical/multi-spectral cameras, and tilt photography. This allows the platform to focus on key areas or emergency scenarios. For instance, it monitors shoreline changes, reservoir floodplains, and the terrain and landform of flood detention and storage areas. It can also track local heavy rain conditions [19]. The UAV performs dynamic monitoring at small scales. This monitoring offers high precision with high temporal and spatial resolution. This capability effectively compensates for the shortcomings of satellite observations by providing better timeliness and more accurate local details.

#### 2.1.3. Land-Based Platform

The land-based platform relies on fixed ground observation facilities. These include hydrological stations, automatic water quality monitoring stations, water level stations, rainfall stations, and video monitoring stations. These facilities are placed at key control sections and sensitive points (such as inflow/outflow outlets, water intake points, and areas near landslide bodies on the reservoir bank). At these locations, the stations conduct continuous, precise, and real-time in situ monitoring. They track core hydrological and water resources elements such as evaporation volume, groundwater level, precipitation amount, and flow rate. They also monitor water quality parameters (such as pH, dissolved oxygen, turbidity, total phosphorus/nitrogen). This process provides essential ground-based reference data [20].

#### 2.1.4. Shipborne Platform

The shipborne platform uses mobile monitoring systems like unmanned surface vessels (USVs). These vessels carry specialized equipment, including acoustic Doppler current profilers, multi-parameter water quality sensors, and depth meters. Their mobility allows them to travel deep into the reservoir or navigate dangerous waters. During these voyages, they conduct high-precision, in situ measurements of the reservoir’s water dynamics and environment. For example, they track water level, flow velocity, and water depth topography. They also monitor water temperature stratification, turbidity, conductivity, chlorophyll a, and sediment content. This approach effectively overcomes the spatial limitations of fixed, shore-based observations [21].

#### 2.1.5. Dam Platform

The dam platform deploys sensors directly on the main body of the reservoir dam, important embankments, and other hydraulic structures. It integrates various tools like seepage pressure gauges, distributed fiber optic gratings (for monitoring temperature and strain), strain gauges, inclinometers, displacement meters, vibration monitors, and measurement robots (automatic total stations). This platform provides high-frequency, real-time, and refined perception of core indicators of dam/embankment seepage pressure (pore water pressure), internal/external deformation (subsidence, inclination, horizontal displacement), structural stress and strain states, and vibration characteristics. This system acts as a sentinel for ensuring the safe operation of the dam [22].

#### 2.1.6. Digital Thread Integration

The aforementioned monitoring platforms are connected by a distributed digital thread. This thread covers the “end-edge-cloud” and creates a seamless channel for data. It allows for real-time interaction between multi-platform monitoring devices, edge computing nodes, and cloud-based twin models. This system continuously integrates sensor information into the dynamically updated twin space. This allows for collaborative observations of core elements such as the structural safety status of the reservoir (depending on the dam platform), the dynamic evolution of the reservoir area water conditions (depending on satellite, UAV, land-based, and shipborne platforms), and changes in the water environment (depending on shipborne and shore-based platforms). The system achieves this by coupling multiple physical fields and processes.

### 2.2. Association Processing of Heterogeneous Information

**Figure 3:**
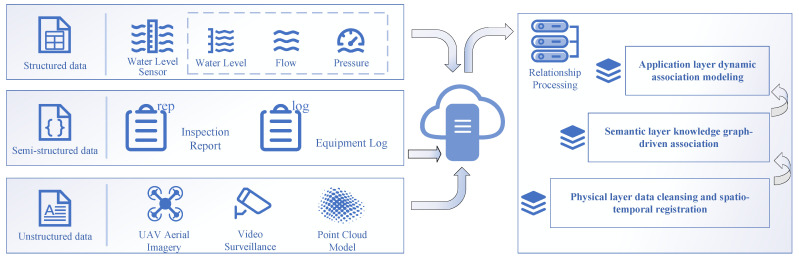
Association processing of heterogeneous information of reservoirs.

In a digital twin system for water conservancy, heterogeneous information mainly manifests as the multi-dimensional heterogeneity of multi-source data [23]. First, data format heterogeneity encompasses structured data (such as sensor time-series signals), semi-structured data (such as inspection report texts), and unstructured data (such as drone aerial images and 3D point clouds). Secondly, semantic hierarchical heterogeneity is reflected in the cognitive differences in cross-domain terms, for example, the “permeable pressure” parameter in the engineering field and the “pore water pressure” indicator in the hydrological model; although their physical essence is the same, there are expression barriers in professional contexts. Thirdly, spatial-temporal scale heterogeneity is prominently manifested as the mismatch in time and space resolution between microstructure monitoring data (millimeter-level displacement sampling frequency) and macro-scale watershed hydrological data (hourly update cycle). These heterogeneous characteristics lead to the data island effect, forming a typical predicament of “data rich but knowledge poor”, and urgently require the establishment of a systematic correlation mechanism (Figure 3). Therefore, this study proposes to construct a “physical-semantic-application” three-layer correlation architecture.

The physical layer achieves the temporal-spatial alignment of heterogeneous data through a unified time-spatial reference framework [24], using time-spatial encoding, data interpolation/aggregation techniques to bridge the scale gap between micro-sensor time-series data and macro-scale watershed models, and establishing a temporal-spatial correlation bond for cross-source data. For structured data such as time-series data of water level, flow rate, and pressure collected by sensors, real-time access and storage are achieved through standardized interfaces [25]. For semi-structured data, such as inspection reports and equipment logs, natural language processing (NLP) technology is used for information extraction and structuring. For unstructured data, such as drone aerial images and video surveillance, key features are extracted using computer vision and image recognition technologies to achieve content tagging and indexing.

The semantic layer relies on domain ontology models and knowledge graphs to build semantic hubs [26]. It maps and defines the concepts and relationships of cross-domain terms such as “permeable pressure” and “pore water pressure”, eliminating the semantic barriers between structured, semi-structured, and unstructured data, and achieving precise anchoring of physical parameters and business concepts. In the ontology model design, a unified terminology and concept framework is established to solve the semantic differences between different data sources. It ensures the consistency and comprehensibility of data. A knowledge graph is constructed to integrate structured and unstructured data, establishing a knowledge graph in the water conservancy field, defining entities (such as “permeable pressure” and “pore water pressure”) and their relationships, and achieving semantic correlation across data types.

The application layer is oriented toward specific business scenarios, building a flexible and scalable business-driven application system to achieve efficient conversion of multi-source heterogeneous data to decision support [27]. For example, in flood control scheduling, combining real-time monitoring data and historical cases, using simulation engines to predict flood evolution, and supporting multi-scheme scheduling decisions. In engineering diagnosis, integrating structural health monitoring data and inspection image information, using artificial intelligence algorithms for anomaly identification and risk assessment, and achieving an automated closed-loop from perception data to diagnostic conclusions. Additionally, the visualization platform can be used to display the real-time status and analysis results of various data, improving users’ perception and decision-making efficiency.

### 2.3. Cross-Model Coupling for Decision Support

Effective management of water monitoring systems requires the collaborative integration of core aspects, including water safety, resources, environment, and ecology [28,29]. For water safety, deformation and seepage monitoring equipment is used to track the stability of structures in real time. Data analysis from this equipment helps to warn against the risks of dam breaks and leakage. Water resource management relies on hydrological monitoring systems, such as radar water level/flow meters and rain gauges. These systems dynamically monitor the hydrological elements of the river basin. This data, combined with intelligent algorithms, allows for precise water volume scheduling [30]. To govern the water environment, water quality monitoring systems (pH, ammonia nitrogen, and COD analyzers) and floating sensor networks track pollution sources. Models then use this data to evaluate and formulate governance plans [31]. Water ecological protection integrates chlorophyll and algae density data with information from UAV inspections. This helps analyze ecological degradation trends and generate restoration strategies. In each of these areas, data are aggregated through collection units, communication networks, and intelligent inspection terminals. This information flows into a comprehensive monitoring platform that provides multi-dimensional decision support, highlighting the systematic nature of digital water resource management.

**Figure 4:**
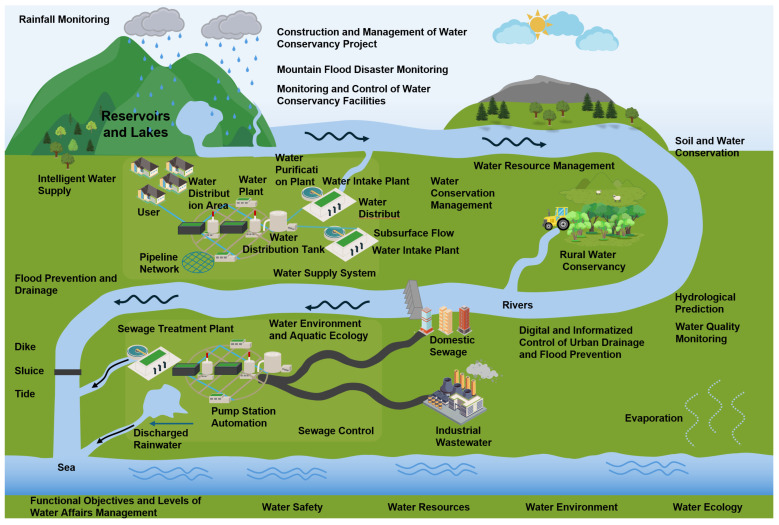
Coupled-model application in reservoir management decision-support.

Effective water system management requires an integrated approach across multiple operational dimensions (Figure 4). These include water safety, resources, environment, and ecology. This process must integrate multiple models, such as hydrological, hydrodynamic, water quality assessment, risk prediction, and optimization scheduling models. Different tasks require different model combinations. For example, rainfall forecasting requires a meteorological-hydrological coupling model [32]. Flood evolution relies on multi-dimensional hydrodynamic models [33]. Reservoir scheduling combines mechanism-driven reservoir scheduling algorithms with reinforcement learning, and water quality management integrates physical migration models with data-driven pollution source tracing models. A single model is limited to local processes and cannot achieve multi-objective collaboration across the entire basin. This is especially true in extreme scenarios. In such cases, it is necessary to simultaneously solve cross-temporal coupling problems, such as the transmission of meteorological forecast errors, the lag effect of engineering scheduling, and the dynamic assessment of socioeconomic losses. To balance multiple goals like flood control, water supply, and ecology, multi-objective optimization algorithms are needed. Through integration with a simulation-based feedback loop, these algorithms support continuous optimization of model parameters and weighting factors.

### 2.4. Closed-Loop Management System for All-Scenario Interconnection

**Figure 5:**
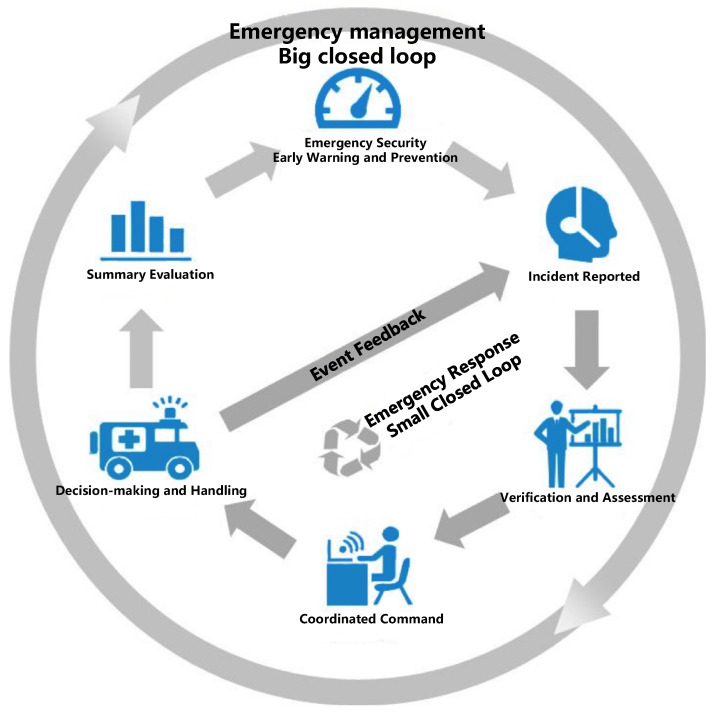
Reservoir management loop, small loop, and large loop.

The main scenarios involved in reservoir management include daily operation, flood control and drought relief, sudden emergencies, ecological environment, engineering construction, and public services [34]. Effective management requires a complete loop, as shown in Figure 5. This loop covers “early warning/prevention/emergency support, event reporting, verification and analysis, decision-making and handling, event handling, summary and evaluation,” encompassing the entire process from risk monitoring, real-time response to post-event optimization [35]. Linking these scenarios is essential because they exhibit close temporal and logical correlations. For example, early warning and prevention should be based on forecast data and deployed in advance. After event reporting, rapid verification and analysis are needed to support scientific decision-making. Emergency handling requires coordinated command and multi-party resource collaboration. Additionally, summary and evaluation rely on feedback from all links to optimize the plan. A lack of cross-scenario linkage will lead to information islands, delayed responses, and resource mismatches, making it difficult to achieve the goal of full-cycle risk resilience governance and multi-modal emergency response coordination in water conservancy management.

The four steps of forecasting, warning, rehearsal, and planning constitute a systematic prevention and control mechanism for responding to emergencies and risks. That is, through early perception (forecasting), timely notification (warning), simulation practice (simulation), and scientific handling (planning), the reservoir operation risks can be actively prevented and efficiently responded to. The integrated multi-sensor information system supports the “forecasting—warning—simulation—planning” closed-loop of water conservancy management through virtual-real mapping and dynamic simulation [36]. In the forecasting stage, the system integrates meteorological, hydrological, and engineering sensor data to construct a panoramic model of the river basin and predict risks such as floods and droughts in real time [37,38]. In the warning stage, based on threshold analysis and simulation results, automatic triggering of graded warning signals is initiated and pushed to the emergency command platform. In the simulation stage, through the integrated multi-sensor information system engine, the evolution path and disposal plan of disasters are simulated to evaluate the feasibility and effectiveness of different plans. In the planning stage, relying on the rehearsal conclusions and historical case library, emergency strategies are dynamically generated or adjusted to form a “forecasting-warning-simulation-planning” intelligent closed-loop. The system achieves seamless linkage from risk perception to action feedback through multi-source data fusion, model-driven decision-making, and cross-departmental collaborative interfaces. It comprehensively enhances the proactive defense and precise governance capabilities of water conservancy management.

## 3. Results

The Ye Fan Reservoir is located in Jingshan City, Hubei Province, China, approximately 50 km from the city center. The main project of the Ye Fan Reservoir includes a main dam, two auxiliary dams, high and low water conveyance pipes, an overflow dam, and a clear water channel. The main dam is a homogeneous soil dam with a height of 20.28 m, a length of 422 m, and a top width of 6 m. The high-water conveyance pipe is a reinforced concrete circular pressure pipe with an inner diameter of 1.5 m, and the water conveyance flow rate is four cubic meters per second; the low water conveyance pipe is a prefabricated pipe with an inner diameter of 0.5 m. The total storage capacity is 1244 million cubic meters, and the effective storage capacity is 822.1 million cubic meters. The irrigation area is 28,000 mu (approximately 1866.7 hectares), and the reservoir’s aquaculture area is 18.8 square kilometers.

**Figure 6:**
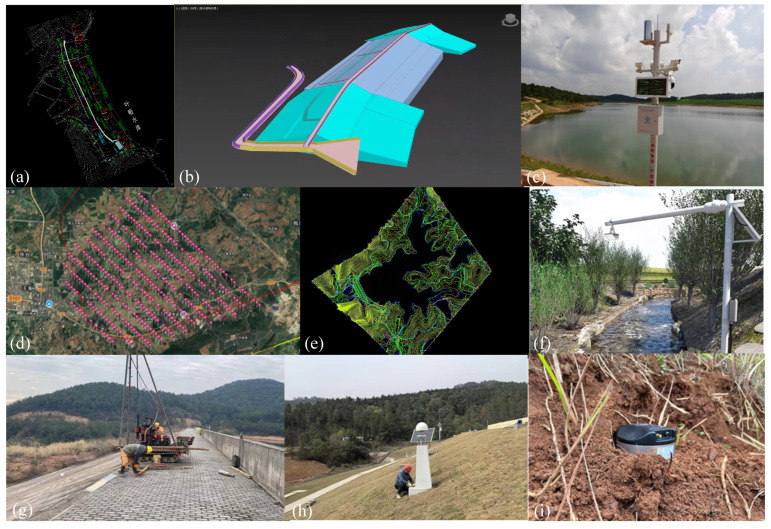
Data collection for Ye Fan Reservoir monitoring. (**a**) dam Computer Aided Design (CAD) design drawing; (**b**) dam Building Information Modeling (BIM) model; (**c**) video monitoring; (**d**) Unmanned Aerial Vehicle (UAV) mapping sampling points; (**e**) Digital Elevation Model (DEM) around the reservoir; (**f**) water level monitoring; (**g**) installation of dam body piezometer; (**h**) Global Navigation Satellite System (GNSS) observation points; (**i**) termite monitoring.

From August 2024 to May 2025, we implemented and validated an integrated monitoring and management framework for the reservoir using this site as a pilot implementation. In the project, a multi-level and all-element perception network and an intelligent equipment system were constructed, as shown in Figure 6. At the end of the data collection, the dam safety monitoring system deployed 27 sets of GNSS satellite receivers and their corresponding antennas, covering the main dam, auxiliary dam, and key geological structure areas. Additionally, 77 seepage pressure gauges and two water level gauges were deployed to achieve precise monitoring of deformation and seepage. The hydrological automatic reporting system was equipped with three rain gauges, one water level station, and two flow stations. It was also equipped with radar flow meters and ultrasonic water level meters to obtain real-time data on the inflow/outflow, rainfall, and wind speed and direction in the reservoir area. The video monitoring system was powered by both grid electricity and solar energy. It included 22,400 W high-definition infrared cameras and panoramic cameras to achieve 24 h continuous monitoring and intrusion alarm for key areas such as spillways and gates. In addition, the termite online monitoring system constructed a dynamic perception network through 170 underground intelligent monitoring devices. The water quality monitoring system integrated floating multi-parameter analyzers, chlorophyll sensors, etc., to conduct in situ real-time detection of water quality in the reservoir area. The total station, UAV inspection system, and satellite emergency communication equipment provided high-precision positioning and remote communication guarantees for manual inspection and emergency response.

**Figure 7:**
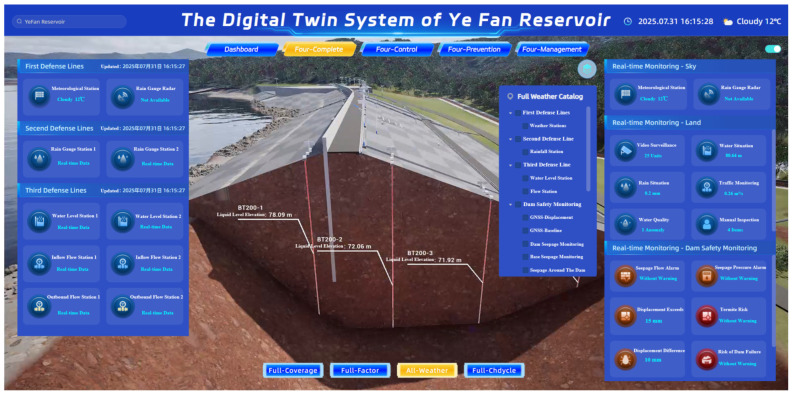
Snapshot of the integrated reservoir management system for the Ye Fan Reservoir dam, showcasing its dynamic visualization capabilities.

The integrated multi-sensor information system developed in this study effectively combines monitoring data with operational applications. A representative interface of the system is presented in Figure 7. The platform is based on BIM 3D modeling, tilt photography, and geographic data. It integrated L2/L3-level models of facilities such as the main dam and spillway. Through the data platform, it realizes the spatio-temporal integration of monitoring data and business data. The model library is equipped with professional algorithms such as water tank models and flood optimization scheduling models, supporting flood evolution simulation and pre-performance of scheduling plans. The intelligent application layer covers three core functions: “one map” comprehensive display, intelligent flood scheduling, and engineering safety warning. Among them, the “one map” system integrates real-time data, including water and rainfall conditions, video surveillance, and reservoir capacity and water level. It supports the drawing of rainfall on surfaces and three-dimensional situation simulations. The safety monitoring and warning module uses dam displacement threshold analysis, termite activity identification, and water quality anomaly detection to achieve multi-source risk multi-level linkage alarms. The system also includes a mobile application, providing inspection trajectory tracking, warning work order handling, and monitoring data reporting functions. It relies on 11 types of security devices, including firewalls, intrusion prevention, and log auditing, to build an active defense system that ensures data security throughout its life cycle. Through the digital integration of the “forecasting-warning-simulation-planning” four-prevention mechanism, the system significantly improves the precision level of reservoir flood control scheduling, safety operation, and emergency decision-making.

The integrated multi-sensor information system of Ye Fan Reservoir achieved real-time dynamic monitoring of the reservoir’s operating parameters and full-cycle data flow management through the construction of a multi-dimensional and high-density perception network. Figure 7 demonstrates the high-fidelity visual representation of the Ye Fan Reservoir dam, showcasing not only its physical authenticity but also its capability to support critical operational functions and business analytics in a virtual space. The system utilizes pressure-type water level gauges, radar water level gauges, and flow velocity area sensor methods to collect data on water level, flow rate, and rainfall at a minute-level frequency for water and rainfall monitoring. The data is transmitted back in real time using the Beidou satellite and a 5G dual-channel transmission mechanism. The water level monitoring stations continuously track changes in reservoir capacity.

**Figure 8:**
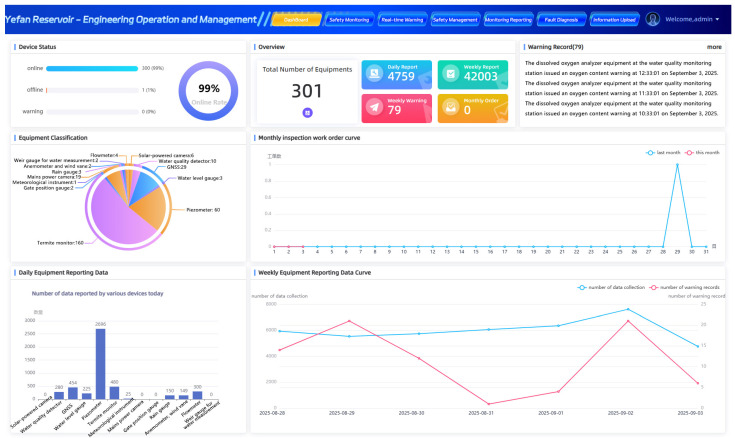
Integrated multi-sensor network platform for real-time monitoring and operational visualization in Ye Fan Reservoir management system.

Figure 8 demonstrates the IoT monitoring information of Ye Fan Reservoir. When the water level exceeds the flood limit threshold, a graded warning is automatically triggered. The dam safety monitoring module uses GNSS receivers and seepage pressure sensor networks to capture millimeter-level displacements and seepage field changes in the main and auxiliary dams in real time. Combined with a three-dimensional monitoring matrix formed by 27 GNSS stations and 77 seepage pressure sensors, deformation data is updated in seconds, and abnormal fluctuations are identified. The video monitoring system utilizes intelligent analysis algorithms to automatically detect events, including personnel intrusion in the spillway area and abnormal gate displacement, within a 1705-camera panoramic network. Through infrared thermal imaging and pan-tilt linkage technology, night-time working conditions can be visualized.

The system’s online rate remains above 90%. All real-time data is pre-processed by edge computing nodes and aggregated to the cloud data lake through the Message Queuing Telemetry Transport (MQTT) protocol. In the data cleaning engine, outliers are removed, and data normalization is performed. Finally, the Reservoir’s “One Map” cockpit enables comprehensive 3D situational analysis. It dynamically renders rainfall isotherms and updates gate status in real-time, providing simultaneous access to both physical site data and virtual mappings for management decision-making. The system has also established a multi-level linkage early warning mechanism. When the water level exceeds the limit, seepage suddenly changes, or equipment is offline, both audible and visual alarm devices are triggered for on-site warning. Work orders are pushed to the person responsible via the mobile terminal, forming a closed-loop response chain from the perception layer to the decision-making layer.

**Figure 9:**
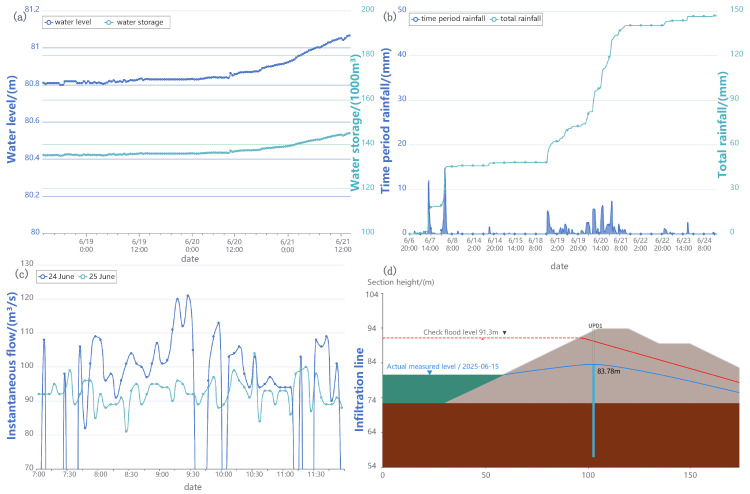
Real-time measurement data of the reservoir/dam ecological monitoring system. (**a**) Reservoir storage capacity and water level; (**b**) rainfall situation and total rainfall near the reservoir; (**c**) reservoir flow rate; and (**d**) infiltration line diagram of the dam.

**Figure 10:**
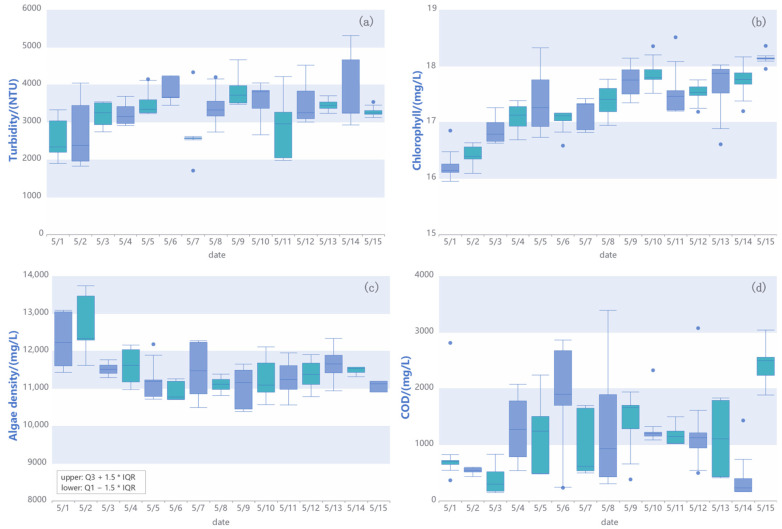
Real-time measurement data of the water quality monitoring system. (**a**) Turbidity, the unit is (Nephelometric Turbidity Unit) NTU; (**b**) chlorophyll; (**c**) algae density; (**d**) Chemical Oxygen Demand (COD).

Figure 9 and Figure 10 present the real-time monitoring data of the reservoir over a period of time. Figure 9a shows the reservoir storage capacity and water level variations, while Figure 9b depicts rainfall patterns and total rainfall near the reservoir. Figure 9c represents the reservoir flow rate over a specific time period, and Figure 9d displays the infiltration line diagram of the dam, highlighting the current measured level and the check flood level. The calculation process of these data is listed in Appendix A.

Additionally, Figure 10 provides data on water quality monitoring, including turbidity, chlorophyll, algae density, and chemical oxygen demand (COD), with box plots to indicate variations over time. These real-time monitoring data provide a continuous, stable, and reliable data source for people to dynamically and intelligently monitor the operating status of the reservoir. These data serve as the foundational real-time inputs for the integrated multi-sensor information system.

We adopted the four-layer architecture presented in Section 2 to manage the diversity of “perception,” “data,” “model,” and “application.” Standardized interfaces ensure interoperability between the layers. The perception layer employs protocols like MQTT and RESTful APIs to flexibly integrate heterogeneous devices. The data layer utilizes open-source databases such as PostgreSQL, MongoDB, and Redis to manage structured and unstructured information. At the model layer, open AI frameworks such as DeepSeek enable seamless integration of different analytical and predictive models. The application layer is supported by containerization and distributed computing tools, including Docker, Apache Spark (Version: 3.5.1), and Kubernetes (Version:1.20), together with Cesium JS and WebGL for scalable deployment and customizable visualization. This modular, open-source design is highly adaptable. The platform is not limited to reservoirs and can be tailored for hydrological, environmental, and other infrastructure contexts.

Compared to existing international practices, this study’s unique contribution is its application of digital twin technology in the Chinese context. It combines a multi-sensor monitoring network with a multi-layer early-warning system. This integrated framework is well-adapted to the complexity of reservoir management in China. It also provides a valuable reference for similar large-scale water infrastructures worldwide.

Following the establishment of the integrated multi-sensor information system for Ye Fan Reservoir, we conducted an evaluation of the improvements in related operational processes and quantified key performance indicators, as presented in Table 1. This research significantly improved the intelligence level and operational efficiency of reservoir management through the construction of a digital twin system. Following system implementation, data transmission latency was reduced from the hourly scale to near real-time. The error rate of flood forecasting, previously based on historical data, was reduced from 20 to 30% to below 10%, the emergency response time was shortened from 2 h to 10 min, and flood regulation achieved response times within seconds. Additionally, annual maintenance costs were reduced from 500,000 yuan to 200,000 yuan. The system integrates multi-source sensor technologies, including GNSS, piezometers, and stress meters, to enable real-time monitoring of dam safety. It also supports 48 h flood forecasting and AI-based video behavior recognition. Leveraging the integration of BIM and GIS technologies, the system enables dynamic simulation of emergency response plans, as well as real-time monitoring and early warning capabilities via mobile devices, thereby comprehensively enhancing the precision and responsiveness of reservoir management.

## 4. Conclusions

This study demonstrates that an integrated multi-sensor information system, leveraging real-time dynamic monitoring, multi-source data integration, and analytical decision support, can significantly enhance the operational efficiency, risk preparedness, and management safety of water conservancy facilities. It supports the transition of water management practices toward data-informed precision. We proposed and implemented a four-layer “perception–data–model–application” architectural framework for reservoir monitoring, which was successfully deployed and validated at Ye Fan Reservoir in Hubei Province. The system integrates multi-source heterogeneous data and cross-scale modeling techniques to establish a full-element dynamic monitoring system spanning from microscopic structural behavior to macroscopic hydrological processes.

Architectural diagram of the integrated reservoir monitoring and information system framework is proposed. It integrates collaborative observations from multiple platforms, processes heterogeneous information, enables decision support through cross-model coupling, and provides closed-loop management. At the collaborative observation level, it integrates multi-source equipment such as satellite remote sensing, unmanned aerial vehicle inspection, GNSS displacement monitoring, and seepage sensors to form a three-dimensional perception network covering all elements of the reservoir. It achieves dynamic collection of millimeter-level deformation and macro-hydrological data. For multi-source heterogeneous information, it proposes a “physical-semantic-application” three-layer correlation architecture. It uses time-spatial registration, knowledge graphs, and dynamic modeling technologies to solve the problem of data heterogeneity and complete the transition from data cleaning to knowledge reasoning. At the model coupling level, it designs a cross-domain collaborative mechanism to dynamically link hydrological, hydrodynamic, and water quality models with multi-objective optimization algorithms. It achieves comprehensive decision-making for multiple objectives such as flood control, water supply, and ecology. In addition, a “forecasting-warning-simulation-planning” full-scenario closed-loop management system is constructed. This system supports the full-process linkage of risk prediction, hierarchical warning, scheme pre-performance, and plan optimization. This is achieved through virtual-real mapping and dynamic simulation technologies, significantly enhancing the precision level and emergency response capability of reservoir management.

Future research can expand upon this work in several key directions. One focus is model fusion refinement. Researchers should explore high-fidelity simulation algorithms for multi-physical field coupling. This will improve prediction accuracy, especially under extreme conditions. Another direction is autonomous intelligent decision-making. Technologies like reinforcement learning and causal reasoning could enhance the adaptability of the system in dynamic environments. The application scope can also be broadened. This includes emerging scenarios such as ecological flow regulation and the protection of cultural heritage within reservoir areas. Additionally, developing open system architectures and establishing digital twin standards for the water conservancy sector will be essential. This will promote cross-platform data and model interoperability. The role of heterogeneous sensors and sensing technologies will be critical. For example, sensor-driven digital twin systems could incorporate new indicators for carbon footprint monitoring and ecological risk assessment. This can help construct a new paradigm of smart water conservancy that balances engineering safety with ecological sustainability.

## Figures and Tables

**Table 1:** Efficiency improvement situation after the integrated multi-sensor information system was completed.

Indicator	Before Implementation	After Implementation
Data delay	>1 h	Near real-time.
Flood forecasting error rate	20–30%	<10%
Emergency response time	2 h	10 min
Flood discharge regulation time	30 min	Seconds
Annual maintenance cost	500,000 RMB	200,000 RMB
Dam safety monitoring	Manual inspection	Comprehensive monitoring via GNSS, piezometers, and stress gauges, with real-time data transmission.
Flood forecast	Experience formula	48 h forecast capability.
Video surveillance	Manual inspection	Panoramic monitoring integrated with AI-powered behavior recognition.
Emergency plan	None	Integration of BIM and GIS for real-time simulation and analysis.
Mobile application	None	Real-time data query, remote control, and warning push notifications.

## Data Availability

The original contributions presented in this study are included in the article. Further inquiries can be directed to the corresponding author.

## Appendix A

Figure 9a:

In reservoir capacity computation, the trapezoidal method and the frustum volume method are among the more accurate approaches available. The model begins by assessing the morphological characteristics of the reservoir to determine whether the trapezoidal formula (Formula (A1)) or the frustum formula (Formula (A2)) is more appropriate. The water column is then discretized horizontally into multiple layers along the depth direction. Each layer is treated as either a trapezoidal prism or a frustum, and its volume is computed sequentially using the frustum volume formula. Finally, the total reservoir capacity (Formula (A3)) is derived by summing the volumes of all individual layers [39].

(A1) $$V_{i}=\frac{1}{2}\Delta h(S_{i-1}+S_{i})$$

(A2) $$V_{i}=\frac{1}{3}\Delta h(S_{i-1}+{\sqrt{S_{i-1}S_{i}}+S}_{i})$$

(A3) $$V=\sum_{i=1}^{n}V_{i}+V_{0}$$

where V represents the total reservoir capacity (water storage), $V_{i}$ denotes the storage capacity of the *i*-th layer, and $V_{0}$ refers to the dead storage of the reservoir, all measured in m^3^. $S_{i}$ indicates the surface area of the *i*-th water layer in m^2^, typically obtained through topographic surveys conducted before reservoir construction. Δh corresponds to the water level difference between the *i*-th and (*i*−1)-th layers, expressed in meters, which can be monitored using water level sensors.

Figure 9b:

(A4) $$R_{t}=\sum_{i=1}^{n}R_{i}+R_{0}\;$$

where $R_{t}$ represents the total rainfall near the reservoir, measured in mm; $R_{i}$ denotes the rainfall amount during the *i*-th time period, which is obtained from rain gauge sensors, also in mm; and $R_{0}$ indicates the initial cumulative rainfall.

Figure 9c:

(A5) Q=Sv

where Q represents the instantaneous discharge, measured in m^3^/s; *S* denotes the cross-sectional area of the reservoir outlet, in m^2^, which is estimated via 3D reconstruction; and vindicates the instantaneous flow velocity, in m/s, as measured by a flow meter installed at the outlet.

Figure 9d:

The phreatic line plot is used in the analysis of groundwater level variations, soil stability, reservoir seepage, and other related phenomena. For subsurface flow, it satisfies the following two-dimensional steady seepage equation:

(A6) $$\frac{\partial }{\partial x}\left(K_{x}\frac{\partial H}{\partial x}\right)+\frac{\partial }{\partial y}\left(K_{y}\frac{\partial H}{\partial y}\right)+Q=\frac{\partial \theta }{\partial t}$$

where *H* represents the function of the total water head, in meters (m); $K_{x}$ and $K_{y}$ denote the hydraulic conductivity in the *x*- and *y*-directions, respectively, expressed in m/s; *θ* is the volumetric water content, dimensionless; and *Q* represents the source/sink term, with a unit of s^−1^ [40].
